# On the contributions of topological features to transcriptional regulatory network robustness

**DOI:** 10.1186/1471-2105-13-318

**Published:** 2012-11-30

**Authors:** Faiyaz Al Zamal, Derek Ruths

**Affiliations:** 1 School of Computer Science, McGill University, Montreal, Canada

**Keywords:** Robustness, Scale-free, Topology, Transcriptional network

## Abstract

**Background:**

Because biological networks exhibit a high-degree of robustness, a systemic understanding of their architecture and function requires an appraisal of the network design principles that confer robustness. In this project, we conduct a computational study of the contribution of three degree-based topological properties (transcription factor-target ratio, degree distribution, cross-talk suppression) and their combinations on the robustness of transcriptional regulatory networks. We seek to quantify the relative degree of robustness conferred by each property (and combination) and also to determine the extent to which these properties alone can explain the robustness observed in transcriptional networks.

**Results:**

To study individual properties and their combinations, we generated synthetic, random networks that retained one or more of the three properties with values derived from either the yeast or *E. coli* gene regulatory networks. Robustness of these networks were estimated through simulation. Our results indicate that the combination of the three properties we considered explains the majority of the structural robustness observed in the real transcriptional networks. Surprisingly, scale-free degree distribution is, overall, a minor contributor to robustness. Instead, most robustness is gained through topological features that limit the complexity of the overall network and increase the transcription factor subnetwork sparsity.

**Conclusions:**

Our work demonstrates that (i) different types of robustness are implemented by different topological aspects of the network and (ii) size and sparsity of the transcription factor subnetwork play an important role for robustness induction. Our results are conserved across yeast and *E Coli*, which suggests that the design principles examined are present within an array of living systems.

## Background

Robustness to evolutionary and environmental perturbations is widely regarded as an important feature of living systems [1]. Despite this fact, much is still unknown about the mechanisms through which robustness is achieved in an organism’s subsystems. In this paper we consider this question within the context of transcriptional regulatory networks, the biochemical systems responsible for controlling the transcription of genes into RNA in response to activating or repressing inputs from transcription factor (TF) molecules. In such systems, one form of robustness is the network’s ability to retain functionally equivalent RNA expression levels when the network is subjected to significant perturbations [2]. Such robustness is important if only because stochastic evolutionary processes and environmental variability frequently introduce small perturbations which can impact the concentration of transcription factors, nutrients, and other biochemical molecules. Robust mechanisms can accommodate these local and temporary changes without compromising the functionality of the overall transcriptional program. Numerous studies on different regulatory networks have established their robustness to mutations and environmental fluctuations (e.g., [3-8]).

While unveiling the exact origin of regulatory network robustness is a topic of active research, there is a growing consensus that the structure of the network itself confers a significant degree of robustness, irrespective of the precise biochemical properties of the individual interactions comprising it. This belief is bolstered by the conservation of (1) several large-scale topological properties and (2) certain motifs (local network structures) within transcriptional regulatory networks across an evolutionarily-diverse array of species (e.g., [9-11]). Furthermore, computational studies have confirmed that a variety of topological properties can be associated with or confer some degree of functional robustness: degree distribution, degree assortativity, network motif abundance, and ratios of positive and negative interactions [2,10,12-17]. These studies typically have focused on characterizing how the introduction of a topological property into an otherwise random network (usually either an Erdős-Rényi (ER) or scale-free network) increases or decreases that network’s robustness to certain types of perturbations.

While this approach has yielded significant insights into design principles of robustness, such individual analyses do not permit evaluating the relative contributions of different topological features to the overall robustness of a network. Without such knowledge, it is difficult to rank the relatively major and minor sources of robustness — an important part of understanding the design principles employed by evolutionary processes. To achieve such a comparative perspective, the robustness of each feature of interest must be evaluated within a single framework and, furthermore, the robustness of the overall network of interest (in this case, a transcriptional network) must also be estimated. These are the foci of the present study.

In this paper, we evaluate and compare the contributions made by several individual and combinations of first-order degree-based topological features^1^ to transcriptional network robustness against random perturbation and mutation. In doing so we obtain quantitative insights into the relative robustness conferred by different topological features and, in particular, we demonstrate that the relatively high degree of robustness in scale-free networks is mainly conferred by the relative scarcity of regulatory nodes in such networks. We compare the relative contributions of these features to the structurally-derived topological robustness of two transcriptional networks, *E. coli* and yeast.

It is important to note that we are intentionally conducting this analysis without considering the evolutionary processes that may have produced the features being considered. We have done this in order to approach, as precisely as possible, the question of *how much* robustness is derived from the different degree-based properties, irrespective of how they come to be in the network. Said differently, it is certainly important to know how structures come to be present in a network, but here we are simply interested in characterizing the extent to which structures that are present contribute to the robustness of the network. Adding an evolutionary context to the present study is an exciting and important direction for future work.

In comparing the robustness of different topological features, we make a number of novel findings. First, we obtain strong evidence that robustness against three different types of perturbations often considered in literature (i.e. knockout of genes, parametric perturbation, and initial condition perturbation) are implemented by different combinations of topological features. Second, we show that a transcriptional regulatory system with a small number of regulators acting semi-independently (i.e. cross regulation among regulators is systematically suppressed) is capable of robustly retaining its mRNA expression vector. Furthermore, a substantial portion of the robustness observed in the *E. coli* and yeast transcriptional networks can be explained through limiting the complexity of the overall network and maintaining sparsity of the inter-regulator-links, rather than by imposing a scale-free degree distribution on the network. Finally, we determine that combining the individual topological features considered generally produces significant, but incremental improvements in robustness.

## Results

### Assessing robustness of topological features

The comparison of the robustness conferred by certain topological features required (1) identifying the topological network features to consider, (2) formalizing the types of robustness to consider, (3) developing methods to generate synthetic random networks preserving the topological features of real networks, and (4) establishing a way to compute the robustness of arbitrary directed networks under a model of transcriptional network dynamics. We discuss each design consideration briefly before presenting results. Complete details are available in the Methods section and the Additional file 1.

#### Topological features

We considered three salient first-order degree-based topological properties of transcriptional regulatory networks: (1) transcription factor to target (TF-target) ratio, (2) scale free-exponential (SFE) degree distribution (out-degree follows a power-law, in-degree follows an exponential distribution), (3) suppressed cross-talk among the TFs (TFs have fewer inter-connections than would be expected by chance) [13,18]. These three properties emphasize different aspects of the network’s degree distribution.

Out of these three properties, the SFE property is widely regarded as a robustness inducer as scale free networks have greater resilience to random node removal than unconstrained random networks [9,12,16]. However, Bergman and Siegal [19,20] opposed this view, showing through simulation that degree distribution (scale-free vs. Poisson) is not sufficient to explain the functional properties, including robustness, of regulatory networks. Scale-free topology implies that nodes with small out-degree are more abundant in regulatory networks, which entails that most of the nodes in a transcriptional network have zero out-degree and hence act purely as target nodes of the transcription factors. An inspection of currently available transcriptional network data as well as previous works on transcriptional regulatory network architecture reveals that only a small fraction of genes (about 10%) within the genome act as TFs [21-23]. However, the effect of having such a small TF-target ratio ( $\frac{\text{\# of TFs}}{\text{\# of non-TF genes}}$) on robustness has not been independently studied, which is why we included this property in our analysis. Consideration of TF-target ratio should enable us dissociate its effect from the reported effect of the SFE property.

In addition to relative scarcity, we observe that transcription factors exhibit less inter-connectivity than would be expected by chance, a feature we call *cross-talk suppression*. This can be considered a feature that participates in decreasing the error propagation: having too many inter-connections among transcription factors hurts modular organization and can eventually increase the error propagation between different parts of the network [13]. Available data on transcriptional networks indicate a significant degree of suppression of TF cross-talk, although the observed degree of suppression varies among different datasets [18,23-26]. Table S1 in the supplementary material reports the amount of cross-talk suppression present in different datasets.

As the reference networks for this study, we used published interaction maps of *E. coli* and yeast transcriptional regulatory networks (hereafter called the *reference networks*) [24,25]. Table 1 presents the observed values for the different properties of these reference transcriptional regulatory networks. Although a later version of the Yeast transcriptional network exists [27], the published network map contains only 54% of the identified transcription factors of Yeast, and therefore, it was not used in our analysis. Previous studies have also preferred the use of the first network for the same reason citing ‘much more power to detect significant effects’ [28]. Neither of these two yeast network maps contains the activation or repression profiles of the interactions, which was reported on a much earlier dataset of Yeast [18]. But as that dataset only covered less than 10% of the yeast genes, we decided not to use it for our analysis.

**Table 1:** The observed values of various topological properties in the reference networks

**Property**	**Yeast**	***E.Coli***
Number of Nodes	3458	1680
Number of Edges	8371	4144
Number of Transcription Factors	286	189
Number of Targets	3172	1491
Activator-Repressor Ratio	Not Given	1.113
TF-target Ratio	0.0902	0.1267
Cross-talk Ratio	0.87	0.8344

#### Types of robustness

Closely following prior work, we considered three kinds of robustness: (1) knockout robustness (against the deletion of random nodes in the network), (2) parametric robustness (against changes in the strength of interactions), and (3) initial condition robustness (against changes in the initial transcription factor concentrations) [2,15,29]. Broadly, these model (1) mutations that renders a gene/protein non-functional, (2) mutations that effect the binding strength of the transcription factors to their targets or their effectiveness in recruiting RNA polymerase, and (3) environmental shifts that affect the concentrations of various proteins, nutrients, and gene transcripts, respectively.

#### Synthetic network generation

In order to assess the robustness conferred by a specific single or combination of topological properties, we developed methods for generating networks with those individual or combinations of properties (hereafter, the *target property/properties* of the generative method and its networks). Each generative method was used to produce a set of 1000 networks (called an *ensemble*). The specific values for the target properties of an ensemble were drawn from their respective reference network: e.g., the TF cross-talk ensemble for the yeast reference contained networks that had the same amount of TF cross-talk as in the yeast reference, but had random topology in all other respects. Random weights were assigned to interaction edges, respecting only the activation-repression ratio (the ratio between activating and repressing interactions) of the appropriate reference network. Note that the activation-repression ratio is unknown for the Yeast network. We determined, however, that the choice of activation-repression ratio does not effect the relative ordering of the ensembles based on their robustness and therefore, does not affect the conclusion of our work (see Additional file 1: Figure S2), which is consistent with the finding of Van Dijk et al. in a similar analysis [30]. Thus, we applied the activation-repression ratio of the *E. coli* network to Yeast ensembles as well (our results hold for other reasonable choices of activation-repression ratio as well). Finally, in all cases the size of the network (number of nodes and edges) was set to the size of the reference network.

As our focus is on determining the robustness conferred by first-order degree-based features only, we sought to estimate the level of robustness conferred to the reference networks by all first-order features, discounting any effect of local features (such as motif distribution and local clustering), meso-features such as community structures and higher-order degree-based features (such as degree assortativity). In order to achieve this, we created a shuffled network ensemble where the edges of the reference network were switched to remove any local clustering, keeping all the degree based features invariant. Then we randomly assigned edge weights and initial expression level of the genes keeping to construct a shuffled network ensemble. Networks in the ensemble retain all the first-order degree based features: the three features described as well as the indegree-outdegree-combination (the 2-tuple defining the in and out-degree of a gene) of each gene in the network. The shuffled network ensemble is the directed equivalent to the configuration model random graphs [31] and has been widely used in network randomization literature and network motif-detection tools [10,32,33].

The dynamics of each network in these ensembles were simulated using a standard discrete-time, boolean network dynamics model based on [6]. The state of the network at a given time is the expression state (on/off) of each gene in the network. We observed that almost all networks considered reach a steady state (no change of network state) or a stable oscillatory cycle after a small number of time steps.

#### Quantifying and computing robustness

Robustness of a single transcriptional regulatory network against a specific type of perturbation can be defined as the the probability that a perturbation of that type does not alter the final output state reached by the network (assuming a fixed starting state) [2,15]. Thus, for a given synthetic network and starting state, we compute its robustness by assessing the fraction of perturbations that produce a network which reaches the same final state vector as the unperturbed version. The robustness of an ensemble (and, thus, the target properties it implements) against a perturbation type is the average robustness of all the networks in the ensemble against that perturbation type.

As the networks in an ensemble can originally reach either steady state or oscillatory state, we introduced separate measures of robustness to distinguish these two cases: steady state retention ratio (SRR) and oscillatory-state retention ratio (ORR), respectively. SRR (ORR) of a network originally reaching a steady (oscillatory) state refers to the fraction of perturbations for which the steady (oscillatory) state vector remains invariant even after the perturbation. For a network ensemble and each perturbation type (knockout/parametric/initial condition), we compute the SRR or ORR values for each network contained in it using 100 different random perturbations of the same perturbation type applied to each network within the ensemble. If the network originally reaches a steady state, the SRR of the network is the fraction of these 100 perturbations that produce the same unperturbed steady state vector after perturbation. ORR for a network against a perturbation type can be computed in a similar manner for the networks reaching oscillatory states. It is noteworthy that both SRR and ORR measures of robustness yielded the same results and conclusions presented in this paper.

**Figure 1:**
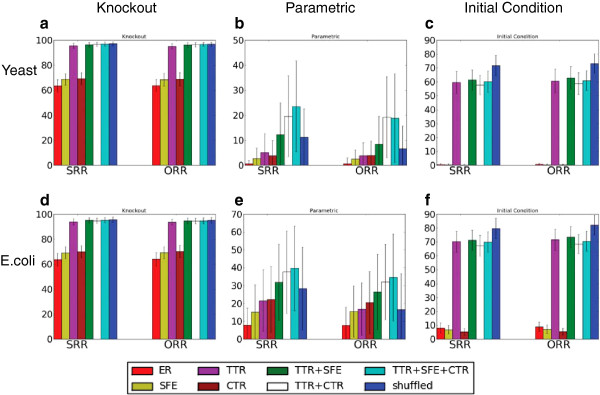
**Robustness of different ensembles.** The Steady State Retention Ratio (SRR) and Oscillation Retention Ratio (ORR) robustness measures for various ensembles. Plots a-c represent random ensembles drawn from the yeast reference and d-f represent ensembles drawn from the *E. Coli* reference. Each bar represents one ensemble and the height of the bar and associated error bar represent the mean and standard deviation, respectively, of the observed SRR/ ORR (robustness) values for the ensemble. Despite numerical differences in the robustness values, both yeast and *E.coli* results consistently show that the transcription factor-target ratio (TTR) and the Cross-talk ratio (CTR) are the most important determinants of robustness, whereas the Scale-free exponential distribution (SFE) is a minor robustness inducer.

The robustness (in terms of SRR and ORR) of different ensembles for different perturbations are reported in Figure 1. Conceptually, we consider each panel to be the *robustness profile* for a given perturbation-reference pair. Each individual bar represents the average SRR (or ORR) value for all networks in the ensemble reaching a steady (or oscillatory) state. The error bars report the standard deviation of the SRR (or ORR) measure for the corresponding ensemble. For knockout perturbations, one node knockout has been considered; for parametric perturbation, the weights have been perturbed by 0.05 (1% of the average edge weight); for initial condition perturbation, 1% of the values in the initial state have been flipped. Note that we have confirmed that the trends we report hold for different perturbation amplitudes, different ways of implementing the underlying perturbations and different definition of robustness.

### Robustness profiles are conserved across species

Comparing the profiles for each perturbation type across species, we observe that the overall shape of the profiles are strongly conserved (e.g., in the knockout profiles in yeast and *E. coli*, Figures 1a and d, the height ordering of the individual properties is the same). To be precise, 96.42*%* of all binary relative robustness score relationships are conserved between yeast and *E. coli* in their SRR profiles for knockout robustness (92.85*%*for ORR profiles). Conservation of relationships are similarly high for parametric robustness (96.42*%* for SRR and 89.28*%* for ORR) and initial condition robustness (92.85*%*for both SRR and ORR).

### Different types of robustness are induced by different combinations of properties

Figure 1 reveals that the three types of robustness considered have quite different robustness profiles, implying that the effects of different kinds of perturbations are blunted by different structural features. Overall, all the three features considered have a positive impact on knockout and parametric robustness (Figure 1a, b, d and e); this is not true of initial condition robustness (Figure 1c and f). This latter profile is particularly striking since robustness only significantly improved under the addition of the TF-target ratio property and the rest of the considered properties had either minor or detrimental effect on robustness. Also of note is the fact that knockout robustness improved most under the TF-target ratio, whereas parametric robustness improved the most when cross-talk was suppressed. All these results strongly suggest that these different kinds of robustness are functions of related, but distinct structural properties.

### Transcription factor-target ratio can explain the robustness effect of scale-free-exponential distribution in regulatory networks

The scale-free topology has been widely acknowledged as a major robustness inducing factor in regulatory networks [9,16,17]. In particular, the presence of hub nodes has been characterized as the key feature inducing robustness in such networks. This view was challenged by Bergman and Siegal [19,20] who demonstrated through simulation that degree distribution does not have a major influence on functional properties of networks, including robustness upon knockout. Our results indicate that a major share of the robustness conferred by scale-free-exponential degree distribution can, in fact, be explained by the relative scarcity of transcription factors (nodes having a non-zero out-degree). Networks not retaining this small TF-target ratio (TTR) property, but retaining the scale-free-exponential (SFE) distribution for other nodes have significantly lower robustness compared to the networks retaining both the TTR and SFE property. The SFE degree distribution does increase knockout and parametric robustness significantly (*p *< 0.001; corrected for multiple testing) compared to the ER networks, but it is significantly lower than the corresponding values observed in the networks retaining the TTR property, which indicates the SFE property is not sufficient to explain the robustness observed in the networks. For initial condition robustness, however, SFE does not increase or decrease robustness significantly. When the SFE degree distribution characteristics is added to a network that preserves other properties, we see a insignificant increase in the knockout robustness. For parametric robustness, the increase is also insignificant for the *E. coli* ensemble that already preserves both the TTR and CTR properties. Overall, the SFE degree distribution property does positively influence robustness to some extent, but its impact is minor compared to that of cross-talk ratio and the TF-target ratio. This finding is consistent with previous work [19,20].

It is worth pointing out that the robustness induction effect of transcription factor to target ratio (TTR) is hardly surprising. A system with a relatively small number of transcription factors will be more robust against random knockout of genes simply because such a random knockout will rarely hit a transcription factor. Similarly, a random change of initial condition affecting only the target genes does not have any impact on the final state reached by the system. However, the novelty of our finding lies in our demonstration that this property can account for a substantial portion of knockout and initial condition robustness that was previously attributed solely to scale-free-exponential distribution.

### Transcription factor-target ratio and suppressed cross-talk are major contributors to robustness

As described above, the TTR and CTR properties are major drivers of robustness in the regulatory networks we studied. For knockout perturbation, both TTR and CTR significantly (*p *< 0.001) improve robustness compared to the ER networks. Furthermore, the networks that retain both these properties induce even greater knockout robustness. For parametric perturbations, CTR is a stronger individual contributor to robustness than TTR or SFE. The introduction of suppressed CTR to a network that preserves the TTR or TTR+SFE properties significantly (*p *< 0.001) boosts the robustness (SRR/ ORR) values for both yeast and *E. coli* networks. Note that the magnitude of impact of cross-talk ratio property differs between the yeast and *E.coli* references. However, the residual effect of the CTR on networks preserving TTR and TTR+SFE properties remain similar. For initial condition robustness, TTR boosts the robustness for both *E. coli* and yeast networks. CTR, on the other hand, significantly decreases robustness when applied to a network that preserves other properties.

**Figure 2:**
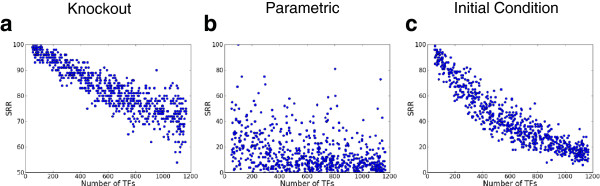
**Effect of the transcription factor abundance on the robustness of*E. coli*ensembles.** The robustness (SRR) values of different networks are plotted against a wide range of the number of transcription factors (TF). All the plots are for an ensemble of 1000 networks where the number of transcription factors has been varied retaining the number of nodes and edges of the *E.coli* reference as constant. SRR values for one node knockout, *α *= 0.05 and *β *= 1% for knockout, parametric and initial condition perturbation have been shown. Increasing the number of transcription factors adversely affects both knockout and initial condition robustness, but does not have a significant effect on parametric robustness.

**Figure 3:**
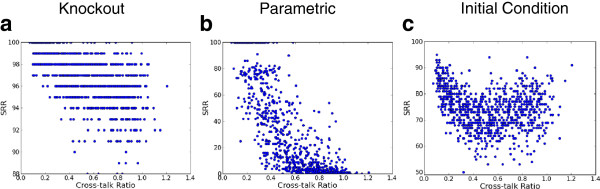
**Effect of cross-talk ratio on robustness of*E. coli*ensembles.** The robustness (SRR) values of different networks are plotted against the Cross-talk ratio. All the plots are for an ensemble of networks with the same number of nodes, edges and transcription factors as the *E. coli* reference where the other topological properties are chosen in random. SRR values for one node knockout, *α *= 0.05 and *β *= 1% for knockout, parametric and initial condition perturbation have been shown. Cross-talk influences all three types of robustness. Increasing cross-talk between transcription factors decreases knockout and parametric robustness. For initial condition perturbations, cross-talk has a dual effect on robustness.

**Figure 4:**
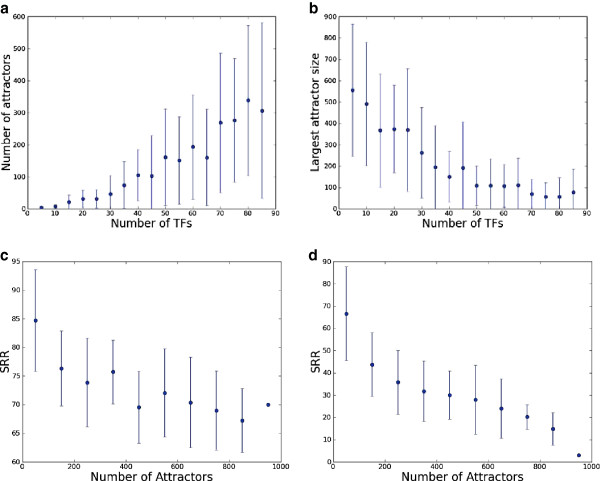
**Increasing the number of transcription factors increases the complexity and decreases robustness.** We trace the steady state attractors of 1000 random networks with 100 nodes and 246 edges each (preserving the node-edge ratio of the *E.coli* network) where the number of TFs have been varied from 5 to 90 for 100 random initial conditions and report the number of attractors (plot **a**) and size of the largest attractor (plot **b**) against the number of transcription factor in the system. As we increase the number of TFs, the number of attractor (and the variance) increases and the size of largest attractor (and the variance) decreases.We also plot knockout (plot **c**) and initial condition robustness (plot **d**), in terms of SRR values, of these networks. Both knockout and initial condition robustness are strongly affected by the number of attractors, although the trend is stronger for the initial condition robustness.

In order to better understand how robustness changed in response to TTR and CTR properties, we evaluated the robustness of networks exhibiting a range of values of TTR and CTR (Figures 2 and 3, respectively). In Figure 2, we see that increasing the number of TFs decreases knockout and initial condition robustness. This trend for knockout and initial condition robustness is due in large part to two interrelated factors. First, a system with a relatively small number of transcription factors reduce the likelihood of a random perturbation hitting a transcription factor node, reducing the impact of such perturbation. More generally, further analysis (Figure 4, Additional file 1: Figures S6, S7) indicates that an increase in the number of transcription factors increases the overall expressive complexity of the network, quantified by measuring the number of attractors in the system [6]. In systems with more attractors, perturbations have a higher probability of reaching different steady-states, decreasing the overall robustness of the system. Nonetheless, while the TTR-complexity-robustness relationship is strong, there is still high variability in the complexity of networks with a fixed TTR, suggesting that the connectivity of networks and the parameter assignments with the same TTR can significantly influence their individual degree of knockout robustness. We identify the relationship among complexity, TTR, and robustness as, itself, a rich area for future work.

In Figure 3, we see that increasing the CTR while leaving other properties unchanged produces an overall decrease of the knockout robustness,a sharp fall for parametric robustness and interestingly, a dual effect for initial condition robustness. For low values of CTR, initial condition robustness is high, which drops off quickly with moderate CTR values. But for higher values, the initial condition robustness increases again. In the case of knockout robustness, if the transcription factors are sparsely connected (i.e. the cross-talk is suppressed) the effect of the deletion of a TF only directly impacts the small neighborhood of the TF. These justify the positive influence of cross-talk suppression over knockout robustness.

In the case of parametric perturbations, densely interconnected transcription factors may amplify a perturbation to an edge weight (there are more neighbor TFs one step away), while abundance of transcription factors (TTR) does not directly render the network more or less susceptible; this explains why CTR is a sole major influencer over this type of robustness.

Under initial condition perturbation, the values of a subset of nodes are being changed in the initial state. A small value of CTR means transcription factors tend to drive genes independently: thus genes are affected by one or a few TFs, which makes these networks more robust against small random perturbations to the initial state. On the other hand, if the transcription factors are highly connected, the effect of changing a gene’s initial state can be neutralized by the impact of other transcription factors, which may explain the dual impact of CTR on initial condition robustness.

It is important to realize, however, that absolute robustness against initial condition perturbation is not desirable because it produces a system that is unable to implement complex input/output relationships (in the extreme case, every input results in the same output). This limits both expressiveness of the transcriptional system as well as adaptability and evolvability [34]. Therefore, it is plausible that suppression of cross-talk is used as a mechanism for trading off between the initial condition robustness and the evolvability of the networks. Furthermore, suppression of cross-talk also gives rise to a modular organization of the transcription factors which promotes autonomy of subsystems - another feature of adaptable and evolvable systems [1,35].

### Exact in-out degree combination observed in real networks reduces parametric robustness

The shuffled network ensemble (rightmost blue bars) preserves all the independently considered first-order degree-based properties as well as the exact combination of in-degree and out-degree of the nodes, a property of the real network which is not preserved in other ensembles (the in-degree vs. out-degree distribution of the reference networks are provided in Additional file 1: Figure S5). As shown in Figure 1(a) and (d), combining the TTR, SFE and CTR properties accounts for the knockout robustness of the shuffled ensemble. This indicates that these three features are sufficient to explain the knockout robustness induced by the global topological features. Furthermore, the in-out degree combination (IOC) does not significantly affect knockout robustness. However, IOC strongly and negatively impacts parametric and initial condition robustness.

## Discussions

This study provides insights into the impact of different first-order degree-based structural features on transcriptional network robustness. To our knowledge, we are the first to consider this question. Our work demonstrates that (i) different types of robustness are implemented by different topological aspects of the network, (ii) size and sparsity of the transcription factor subnetwork play an important role for robustness induction, and (iii) some degree-based features present in real transcriptional networks actually decrease their overall robustness. These conclusions are validated for a discrete time network dynamics model that was previously used to model the dynamics of the budding yeast cell cycle network [6] and close variants of which have been extensively used in similar analysis, e.g. [2,15,16,20,35,36].

### The different topological bases of robustness

All three different types of robustness considered are biologically important. A transcriptional regulatory network should be resilient, at least moderately, against removal of random genes, change in interaction strength due to environmental or mutational effect and initial concentration variation due to environmental shifts. We show that these three types of robustness are engendered by different combination of topological properties and the impacts of a given topological property on three different types of robustness are different. This observation suggests that obtaining one kind of robustness may require a trade-off in terms of another form of robustness. For example, absolute robustness against initial condition perturbation is generally undesirable, for if a network’s output becomes invariant with the change of input, the system loses its functional flexibility. On the other hand, every system should be capable of adapting to small changes due to knockout perturbation. Therefore, the topological features can be evolutionarily tuned to have higher robustness against knockout maintaining an optimal level of initial condition robustness. Future investigations may explore how this trade-off is achieved by evolutionary constraints that shape the system.

### Robustness and sparsity

Prior work has shown that selection favors sparser biological networks to achieve robustness [36]. Our work expands on this finding, suggesting that the robustness in regulatory networks is achieved mainly through a relatively small number of sparsely connected transcription factors regulating a much larger set of target genes. The scale-free-exponential degree distribution property, widely marked in literature as a robustness inducer, has not been identified as the strongest contributor to robustness. Instead, our work shows that a small transcription factor to target ratio, a feature of these scale free networks, can explain a major share of the effect that was supposedly attributed to the scale-free-exponential degree distribution. A system with a small number of regulators acting semi-independently (i.e. cross-talk among regulators is systematically suppressed) is capable of robustly retaining its mRNA expression. While the finding that increasing the number of transcription factor induces a decrease in robustness is rather obvious, the striking aspect of our finding is the amount of robustness that the real systems derive from it, as majority of robustness observed in the *E. coli* and yeast transcriptional networks can be explained through maintaining sparsity of the transcription factor subnetwork and limiting the complexity of the overall network.

### The in-out degree combination diminishes parametric robustness

Quite surprisingly, our results show that for the parametric perturbation, the exact in-out degree correlations present in real transcriptional networks decrease the robustness of those networks to parametric perturbation. Notably, this is not the case for knockout and initial condition robustness: in both cases preserving IOC increases the initial condition robustness compared to all other ensembles. As our goal in this study was to *identify* and *quantify* the relative contributions of different degree-based features to transcriptional network robustness, we leave a thorough investigation into the cause of this correlation for future work. That said, we offer the following hypothesis that explains a mechanism by which IOC could plausibly decrease the robustness of a network.

As the Additional file 1: Figure S5 shows, most of the hub genes (genes with high out-degree compared to the most other genes) in the reference transcriptional networks have moderate in-degree (ranging from 2 to 5) and most master-TFs (genes not regulated by any other gene) have moderate out-degree. To grossly simplify this picture, we can say that real transcriptional networks contain a disproportionate number of low-in/high-out and high-in/low-out nodes. Note that in networks that preserve the in-degree and out-degree distributions, but not the in-out degree correlations of real transcriptional networks, the average out-degree of high in-degree nodes will increase. In such a situation, more edges will terminate in high out-degree nodes, raising the probability that an edge perturbation directly affects a hub and its large downstream neighborhood. We consider this hypothesis a promising starting point for a comprehensive investigation into the unexpected effect of IOC on network robustness.

## Conclusions

Robustness of biological systems against random mutations and environmental perturbations is a widely observed phenomenon. In this study, we assess the relative contribution of first-order degree-based network properties to the robustness of transcriptional regulatory networks. Through extensive simulations, we show that the scale-free-exponential degree distribution, in itself, is a minor contributor to transcriptional network robustness. Much of the effect it exerts can be explained by the relative abundance of target genes compared to transcription factor genes in such systems. Moreover, suppression of cross regulatory edges connecting two transcription factors has a profound impact on the robustness of the networks against certain perturbations. These three properties are sufficient to explain the amount of knockout robustness a transcriptional network derives from first-order degree-based properties; interestingly, the in-degree/out-degree correlations present in real networks account for a non-trivial portion of the parametric and initial condition robustness present.

More broadly, our comparative approach to assessing the robustness conferred by individual topological features and present in reference, real-world networks enables us to ascertain, for the first time, the extent to which different topological properties (and their combinations) induce the robustness observed in these real-world systems. We consider this to be an important and essential step in better understanding the means by which robustness is implemented in transcriptional networks. Our approach may also be applied to the study of robustness in other networks, however they may arise. Thus, while we have applied our approach to transcriptional networks, other domains both within and beyond cellular biology may benefit from the use of such methods on their own complex systems.

## Methods

### Yeast and *E.coli* reference networks

As reference, real-world transcriptional networks, we used yeast and *E. coli* regulatory networks. The *E.coli* regulatory network, consisting of 1680 genes and 4144 interactions, was downloaded from the RegulonDB database (Release: 7.4 Date: March 2012) [24]. The yeast regulatory network subset was taken from the work of Yu et al. 2006 and consists of 3458 genes and 8371 interactions [25].

In these networks, all nodes correspond to genes. Those that regulate (have edges to) other genes are transcription factors (*TF*); all others we call *targets*. In addition, each interaction in the network is designated as being either activating (positive) or repressing (negative). It is important to note that the precise biochemical parameters for interactions are not known for large biochemical networks. As a result, the dynamics of the real and synthetic networks were estimated by generating an ensemble of networks with identical topologies, but parameter values drawn from a distribution.

### Topological features considered

We constructed network ensembles that retained different properties of the reference networks.

#### (1) Transcription factor-target ratio

In a TRN, a gene can code for a transcription factor which regulates other genes. In the network, such genes are simply considered to be the transcription factors themselves (since their expression directly results in an increase in the abundance of the transcription factor). The TF-target ratio is the ratio of the number of TF-coding genes and the number of non-TF genes.

#### (2) Degree distribution

The degree distribution is the allocation of interactions to nodes over the entire network. We consider the in-degree and out-degree distributions separately. For the reference networks, the in-degree distribution follows an exponential distribution but the out-degree is a power-law distribution. We refer to this degree distribution as Scale-Free-Exponential (SFE) degree distribution in the text.

#### (3) Cross-talk ratio

This property refers to the ratio between the observed count of TF-TF interactions to their expected count in an equal sized random network having an equal number of TF-coding genes where edges can be formed independently between a TF as starting point and any gene (either TF or non-TF) as ending point. If *N* and *E* denote the number of nodes and edges in a network and *N*_*TF*_ and *E*_*TF*_ denote the number of TFs and the number of edges connecting two TFs respectively, then the Cross-talk ratio (CTR) will be equal to $\frac{E_{\text{TF}}}{N_{\text{TF}}}/\frac{E}{N}$ which can alternatively be written as $\frac{\langle k_{\text{in}}^{\text{TF}}\rangle }{\langle k_{\text{in}}\rangle }$ where $\langle k_{\text{in}}^{\text{TF}}\rangle$ and 〈*k*_*in*_〉 represents the average in-degree for TFs and for all the nodes respectively.

#### (4) Activation-repression ratio

In a TRN, every interaction (edge) is either activating or repressing. The activation-repression ratio is the ratio of the number of activating edges and number of repressing edges. As the activation-repression information was not reported in the yeast dataset we used, or any other recent datasets [27,37,38], the *E. coli* activation-repression ratio value is used for the yeast ensembles as well. This property, along with the number of nodes and edges of the reference network, was retained in all the network ensembles.

#### (5) In-out degree combination

This property refers to the exact combination of in-degree and out-degree for each of the nodes in a network. Formally, for a node *n*, the in-out degree combination (IOC) of the node is the two-tuple $\langle k_{\text{in}}^{n},k_{\text{out}}^{n}\rangle$ where $k_{\text{in}}^{n}$ and $k_{\text{out}}^{n}$ corresponds to the in and out-degree of the node *n* respectively. The shuffled network ensemble contained networks which retained the IOC for each node in the reference networks.

### Random network ensembles

In order to determine how a specific topological property or a combination of properties influences robustness, we constructed different network ensembles (1000 networks per ensemble) that preserve a different set of properties of the original networks. For each combination of properties, we developed an algorithm that explicitly constrained only the value of those properties in the networks produced. Details for each property and combination considered are given in the Additional file 1. For each network, the strength of the interactions (weights) was randomly assigned from [±1,±9] keeping the average activation-repression ratio equal to the activation-repression ratio of the reference networks.

### Model of network dynamics

We employed a network dynamics model that was used to model the dynamics of the budding yeast cell cycle network [6]. Providing additional support for this model, we independently verified that it is capable of generating oscillatory behavior for the Drosophila circadian clock network [39]. This model assumes a binary expression level for the genes, i.e. genes are either expressed (1) or repressed (0) at any given time. The current state of a gene depends on the total weighted input from its TFs, i.e. the expression level of a gene at time *t* + 1 is dependent on the output of its TFs at time *t* and the weight of the TF interactions on the node. This leads to the use of nonlinear difference equations for modeling the dynamics of regulatory systems [35].

In a regulatory network, the expression level of gene *a* at time *t* + 1, $y_{a}^{t+1}$, is a function of the state of the network at time *t*. We express this as $y_{a}^{t+1}=f(\underset{b\in \text{TF}(a)}{\sum }w_{\text{ba}}\cdot y_{b}^{t})$ where *TF*(*a*) is the set of TFs for *a*, i.e. the set of nodes that have an edge to *a*. We chose the function *f * such that *f*(*x*) = 1 if *x *> 0, *f*(*x*) = 0 if *x *< 0, and $f(x)=y_{a}^{t}$ if *x *= 0.

Thus, a gene is expressed ( $y_{a}^{t+1}=1$) if the sum of the weighted inputs from its TFs is positive, and is not expressed ( $y_{a}^{t+1}=0$) when the sum of the weighted inputs from its TFs is negative. If the total input is zero, the gene retains its expression level at the previous time step.

We added a mechanism for self-degradation in nodes with no inhibitors. If such a node is active at time *t*, but does not have any activating input at that time, then the node output will be set to zero at time *t* + 1.

### Simulating network dynamics and perturbations

For a given network in an ensemble, we simulate the dynamics described above starting from a random assignment of on/off nodes, apply the update rules for up to 100 time steps and record the output values at the final step. If the output of all the nodes remains unchanged for two consecutive time steps during the simulation, we stop our simulation, record the output of the nodes, and mark the parameterized network as having reached a *steady state*. If, instead, we find that the network does not reach a single steady state, but cycles through a set of consecutive states, the network is marked as reaching an *oscillatory* state.

#### Perturbations

For knockout perturbations, we randomly delete one or two nodes from the network; for parametric perturbation, we randomly add ±*α *from the edge weights; for initial condition perturbation, we randomly flip the initial states of a fraction *β* of the total nodes. For each type of perturbation, the dynamics of the networks are simulated on these perturbed networks and the final output is recorded and marked as reaching a steady or oscillatory state. The robustness of a network is its average robustness (in terms of SRR or ORR) to 100 random perturbations (of the same type).

### Basin of attraction analysis

The purpose of the basin of attraction analysis is to ascertain how the number of transcription factors impacts the dynamical complexity of the network. We constructed an ensemble of networks consisting of 1000 networks preserving the average degree of the *E.coli* transcriptional network. Then we applied 1000 random initial state configurations on each of these networks and recorded the output states the networks converge to. The set of states where a network can reach through dynamical simulation defines the attractors of the system and the number of different initial conditions associated with a particular attractor state is an estimation of the size of its basin of attraction of the system. To determine the impact of network complexity on robustness, we also computed the knockout and initial condition robustness (in terms of SRR) for each network in the network ensemble.

### Implementation details

The computational work was implemented in Python. NetworkX was used to load, manipulate, and manage individual networks and Numpy was integral to the implementation of the simulator [40,41].

## Endnote

^1^ By *first-order* degree-based, we refer to features that depend primarily on the degree and linking patterns of the node itself rather than on features that involve analysis of the linking patterns of two or more nodes, such as degree assortativity.

## Abbreviations

TF: Transcription factor; TTR: Transcription factor to target ratio; CTR: Cross Talk Ratio; SFE: Scale-Free-Exponential; IOC: In-out degree combination.

## Competing interests

The authors declare no competing interests.

## Author’s contributions

Both FAZ and DR participated in the study design, analysis of results and writing the manuscript. The network generation and dynamical simulation frameworks were developed by FAZ. Both authors read and approved the final manuscript.

## Supplementary Material

Additional file 1 **Supplementary Information.** In the supplementary information document, we discuss algorithms for generating the synthetic networks conforming to different random network models that have been used in our study and include some additional results to support the claims of our study. Click here for file
