# Supplementary material for: On the contributions of topological features to transcriptional regulatory network robustness
Source: BMC Bioinformatics. 2012 Nov 30;13:318. doi: 10.1186/1471-2105-13-318 (PMC3541983; doi:10.1186/1471-2105-13-318)
Supplement: Additional file 1 — Supplementary Information. In the supplementary information document, we discuss algorithms for generating the synthetic networks conforming to different random network models that have been used in our study and include some additional results to support the claims of our study. [file 1471-2105-13-318-S1.pdf]

Supplementary Information:On the contributions of topological features to transcriptional regulatory network robustness

Faiyaz Al Zamal Derek Ruths

1 Introduction

In this supplementary material document, we discuss algorithms for generating the synthetic networks conforming to different random network models that have been used in our study and include some additional results to support the claims of our study. In Section 2, we discuss the network generation algorithms and in Section 3, we provide the additional results.

2 Generating Different Network Models

We performed a comparative study of the contributions made by different topological features and their combinations on the robustness of transcriptional regulatory network of yeast and *E. coli*. We considered three topological features, i) transcription factor-target ratio, ii) degree distribution, and iii) cross-talk ratio and computed through simulation the robustness induced by the features either taken individually or in collectively with one or more of the other properties. To do this, we need to synthesize networks constrained to preserve only the properties under consideration letting all other properties freely assume their values as they would do in an unconstrained random network. In sections 2.1 - 2.5, we discuss the algorithms for generation of various models.

2.1 Generating Erdős and Rényi (ER) networks

We modify Erdős and Rényi's algorithm for generation of directed version of ER networks [4]. Algorithm 1 describes the procedure for generating an ER network given the number of nodes, edges, activator-repressor ratio and weight range. The algorithm is very simple, it randomly picks two vertices from the set of nodes and joins them if the edge does not already exist. Each edge is assigned a random weight whose magnitude is drawn uniformly from the desired range and the signs are assigned such that the activator repressor ratio of the reference network is preserved. Finally, each of the nodes are assigned an initial state (either 'on' or 'off') randomly. The edge weight range of $[\pm 1, \pm 9]$ was chosen following a related work by Kwon et al [7]. We used the same weight range to parameterize every ensemble in our study. However, the choice of weight range does not affect our conclusions if we appropriately scale the perturbation window parameter α for parametric perturbation.

2.2 Conserving Transcription-factor to target ratio (TTR) property

Algorithm 2 describes the procedure for generating networks preserving the TF-target ratio property. The only difference with Algorithm 1 is that here we first mark a subset of nodes as TFs

Algorithm 1 Generating ER networks

Require: n =number of nodes, e =number of edges, a = activator-repressor ratio, $[w_{min}, w_{max}]$= weight range

1. Create a set of n nodes.
2. Construct the set of edges by repeatedly performing the following tasks.
   (a) Pick a node u randomly as the start vertex of an edge.
   (b) Pick a node v randomly as the end vertex of an edge.
   (c) If the edge $u \rightarrow v$ does not already exist in the graph,
      - Add the edge $u \rightarrow v$ to the graph.

   until the edge set has e elements.
3. For each node, randomly assign the initial state as either on or off.
4. For each edge, assign a random weight ranged from $[w_{min}, w_{max}]$ such that the activator repressor ratio is on an average a.

and then randomly connect the TFs with the nodes. The same principle is applied for constraining the TTR property in other ensembles which preserve further topological properties of the reference networks. In all cases, the edge weights are assigned in the way described in Section 2.1.

Algorithm 2 Generating TF to Target Ratio Preserved Networks

Require: n =number of nodes, e =number of edges, a = activator-repressor ratio, t = TF-target ratio, $[w_{min}, w_{max}]$= weight range

1. Create a set of n nodes.
2. $Set\_TF = \{\}$
3. Randomly choose $\frac{t}{1+t} * n$ nodes and insert them in Set_TF.
4. while number of edge $\leq e$
   (a) Pick a node u from Set_TF randomly as the start vertex of an edge.
   (b) Pick a node v from the set of nodes randomly as the end vertex of an edge.
   (c) If edge $u \rightarrow v$ does not already exist in the graph,
      - Add edge $u \rightarrow v$ to the graph.
5. For each node, randomly assign the initial state as either on or off.
6. For each edge, assign a random weight ranged from $[w_{min}, w_{max}]$ such that the activator repressor ratio is on an average a.

2.3 Constraining the of scale free - exponential (SFE) property

We generate the scale free out-degree property utilizing the Barabási- Albert model of growth and preferential attachment [1] and in-degree exponential property by taking the in-degree count directly from an exponential distribution. Barabási and Albert show that scale free networks emerge from simultaneous operation of growth and preferential attachment, in which new nodes are added to the system causing the network to grow and these new nodes prefer to connect to an already

Algorithm 3 Generating SFE-preserved Networks

Require: n =number of nodes, t =TF-target ratio, λ = Exponential distribution parameter, $[w_{min}, w_{max}]$= weight range, a = activator-repressor ratio

1. Create a set of n nodes.
2. Generate the in-degree count of each node using an exponential distribution having parameter λ.
3. $Set\_TF = \{\}$
4. For each node v, read *in-degree*(v) and construct *in-degree*(v) edges by doing the following:
   (a) if $(|Set\_TF| < \frac{t}{1+t} * n)$,
      - Randomly Pick a node u, connect it to v by adding edge $u \to v$ to the graph,
      - if $u \notin Set\_TF$
        – add u to Set_TF

   (b) else,
      - Preferentially Pick a TF u from Set_TF and connect it to v by adding edge $u \to v$ to the graph.
5. For each node, randomly assign the initial state as either on or off.
6. For each edge, assign a random weight ranged from $[w_{min}, w_{max}]$ maintaining the ratio a of activating weights vs. repressing weights.

well connected node rather than connecting to a node which is not so well connected. This method needs as input the TF-target ratio of the desired network. When we are constraining only the SFE property, the TF-target ratio (TTR) is drawn from the distribution of TTR values observed in an equivalent ER network. When both the TTR and SFE property need to be preserved, the TTR value is set to the observed TTR value in the reference (Yeast or *E.coli*) networks.

Our method is described in details in Algorithm 3. After initializing the set of nodes, we draw the in-degree of each node from an exponential distribution. The exponential λ parameter is set to 0.4103 for Yeast and 0.4026 for *E. Coli*, so that the average number of edges conforms to the number of edges in the reference networks.

For modeling the growth and preferential attachment, we first continue adding edges to the network randomly until we have a set of exactly $\frac{t}{1+t} * n$ nodes who are acting as start vertex, where t is the TF- target ratio of the reference network.

Once we have a complete set of required TFs, we start adding edges using preferential attachment, i.e. we preferentially pick a TF from the TF set with the probability proportional to its existing number of outgoing edges. Mathematically, the probability of choosing a node u from the TF set Set_TF as the start vertex of the next edge is proportional to $\frac{outdegree(u)}{\sum_{v \in Set\_TF} outdegree(v)}$.

After we have constructed our edge set, edge weights and node initial conditions are assigned following the same mechanism as described for ER networks (Section 2.1).

2.4 Preserving the cross-talk ratio

The cross-talk ratio (CTR) of a network is dependent on the in-degree distribution of the transcription factors (TF). In order to preserve the CTR of a network, the in-degrees of the TFs are constrained such that the average in-degree of the TFs is the product of the desired cross-talk ratio and the ratio between the number of edges and number of nodes (i.e. the average in-degree) of

the system. When the CTR is the only property of interest to be preserved, the number of TFs follows a gaussian distribution whose mean and standard deviation is taken from the TF-count distribution of equivalent ER networks. The in-degree distribution of the TFs is then drawn from an exponential distribution that correctly constrains the TF-TF interaction counts. On the other hand, when the transcription-factor target ratio of the reference network is also preserved, the transcription factors were chosen randomly in a similar way described in Algorithm 2. In this case, the TF in-degree distribution is the exact in-degree sequence of the reference networks. The in-degree of the non-TFs are chosen from either a poisson distribution (when SFE property does not need to be preserved) or from an shifted exponential distribution with appropriate exponent (when the SFE property also needs to be preserved).

Algorithm 4 describes the generation mechanism of preserving cross-talk along with both the transcription factor target ratio (TTR) and the scale-free-exponential (SFE) properties. As described above, the in-degree of the transcription factors and non-transcription factors are taken from the TF in-degree sequence of the real reference network and an exponential distribution respectively. The out-degree distribution generation technique is slightly modified from that of SFE preserved network generation. In case of SFE (or TTR+SFE) networks, out-degree distribution is generated by using random attachment followed by preferential attachment. For CT+SFE+TTR networks, however, we have one more attachment mode, which we call equity attachment. In this mode, the first $\frac{t}{1+t} * n$ edges are constructed using each TF exactly once. Once equity attachment is performed, random attachment mode takes over until a pre-defined number of edges P have been constructed. Then, the rest of the edges are added using preferential attachment. The value of P is set such that the expected number of edges added by equity and random attachment mode in these networks is equal to the average number of edges added by random attachment mode in (TTR + SFE) preserved networks, to ensure the out-degree distribution of these two network models are congruent with that of the real network. The motivation behind the use of an equity attachment mode is to make sure that no TF is left without an outgoing edge which is not guaranteed using a random attachment mode only. Likewise, the edge weights are assigned in the way described in Section 2.1.

2.5 Generating the shuffled network ensemble

The shuffled network ensemble preserves the exact in-out degree combination of all the nodes in the network. For the reference *E. coli* and Yeast networks, we randomly pick a pair of edges, switch their endpoints keeping the in-degree and out-degree unchanged (i.e. after shuffling, $A \to B$ and $C \to D$ pairs will become $A \to D$ and $C \to B$) and repeat the switching operations for 20000 times. We generated 1000 different shuffled configuration for each reference network. Like the other ensembles, the nodes are randomly set to initial conditions (on or off) and edge weights are assigned in the way described in Section 2.1.

3 Supplementary results

In this section, we present additional analysis bolstering our conclusions reported in the main paper.

Algorithm 4 Generating Cross talk, SFE and transcription-factor target ratio (CT+SFE+TTR) preserved networks

Require: n =number of nodes, t =TF-target ratio, λ_{non_TF} = Exponential distribution parameter of the non-TF in-degree distribution, $[w_{min}, w_{max}]$= weight range, a = activator-repressor ratio, C =in-degree sequence of TFs, P = Starting point for preferential attachment.

1. Create a set of n nodes.
2. $Set\_TF = \{\}$
3. Randomly choose $\frac{t}{1+t} * n$ nodes and insert them in Set_TF.
4. For each node in Set_TF
   - Generate the in-degree count by random sampling (without replacement) from sequence C.
5. For each node not in Set_TF
   - Generate the in-degree count using a shifted exponential distribution having parameter λ_{non_TF}.
6. $start\_node\_set = Set\_TF$
7. *count_edge*=0
8. For each node v, read *in-degree*(v) and construct *in-degree*(v) edges by doing the following:
   (a) if ($|start\_node\_set| > 0$),
      - In any order, pick a node u from *start_node_set* , connect it to v by adding edge $u \to v$ to the graph,
      - remove u from *start_node_set*.

      else if ($count\_edge < P$),
      - Randomly Pick a node u from Set_TF , connect it to v by adding edge $u \to v$ to the graph,

      else,
      - Preferentially Pick a TF u from Set_TF and connect it to v by adding edge $u \to v$ to the graph.

   (b) *count_edge*=*count_edge* + 1
9. For each node, randomly assign the initial state as either on or off.
10. For each edge, assign a random weight ranged from $[w_{min}, w_{max}]$ maintaining the ratio a of activating weights vs. repressing weights.

Species	Dataset	Number of Nodes	Number of edges	Number of TF	Cross-talk Ratio
Yeast	Guelzim [6]	491	916	110	0.3703
Yeast	Yu [9]	3458	8371	286	0.87
Ecoli	RegulonDB [5]	1641	4144	189	0.8344
Mouse	Bhardwaj [3]	1189	2400	144	0.5848
Tuberculosis	Bhardwaj [3]	756	918	50	0.9058
Rat	Bhardwaj [3]	533	1092	91	0.2413

Table S1: Cross-talk is suppressed in a diverse array of species.

3.1 Cross-talk is suppressed in a diverse array of species.

The Cross-talk ratio of a network represents the ratio between average number of incoming edges to a transcription factor and the average in-degree of the system. For many published dataset of transcriptional regulatory networks, we observed that the cross-talk ratio value is much less than 1.0, the expected cross-talk ratio of a random network if the transcription factors are free to connect to any target, TF or not. In Table S1, we report the cross-talk ratio values for different datasets. While many of these datasets are incomplete, the suppression of cross-talk ratio appears to be a common trait for all these datasets and in all but one case (Tuberculosis), the level of suppression is statistically significant ($p < 0.05$) which establishes cross-talk suppression as a common topological feature existing across a wide array of species.

3.2 Conservation of robustness profile trends across different choice of parameter values and different robustness measures

The trends we report in the paper do not depend on the specific parameter choices and the specific definition of robustness. Instead of requiring exact preservation of output expression vector, if we measure the robustness of an ensemble as the average similarity of the perturbed output expression vector with the unperturbed wild-type output vector, the trends observed in Figure 1 (main paper) still continue to hold. In figure S1, we present the root mean squared deviation (RMSD) distribution of the perturbed output expression vector from the unperturbed vector for all the ensembles against two different perturbation magnitude per perturbation type. The RMSD between two vectors $\mathbf{x}$ and $\mathbf{x}'$, both having length n is calculated using the traditional formula: $RMSD(\mathbf{x}, \mathbf{x}') = \sqrt{\frac{\sum_{i=1}^{n}(x_i - x_i')^2}{n}}$. If $\mathbf{x}$ and $\mathbf{x}'$ correspond to the steady-state output vectors of the unperturbed and perturbed network, respectively, then n is the number of nodes in the network and the RMSD will assess the average amount by which the node outputs deviates upon a perturbation, while the state retention ratios (SRR and ORR) manifest how robustly a network can retain its precise functionality. A higher value of RMSD signifies more deviation from the wild type upon perturbation and hence, less robustness. As Figure S1 reveals, although changing the extent of perturbation obviously scales the specific RMSD values, the relative ordering remains preserved for almost all cases, exhibiting that the trends we report are not products of specific choice of parameters and arbitrary definition of robustness.

3.3 Relative robustness trends remain invariant for different activation-repression ratio

Previous work has shown that the activation-repression ratio considerably impacts the robustness of a network against random perturbations [8]. In order to analyze how the robustness profile is influenced by the change of activation-repression ratio, we generated different ensembles of networks that are topologically identical, but differs only in their activation-repression ratio. Our results suggest that although changing activation-repression ratio influences the absolute value of the SRR and ORR against parametric and initial condition perturbations, the relative ordering of the ensembles in terms of robustness remains generally invariant. For example, 100%, 96.43% and 92.85% of the pairwise relationships for SRR for knockout, parametric and initial condition robustness respectively remains unchanged when the activation-repression ratio is changed to 1.0 from 2.0. Figure S2 presents the robustness profiles for different values of activation-repression ratios for all the network ensembles.

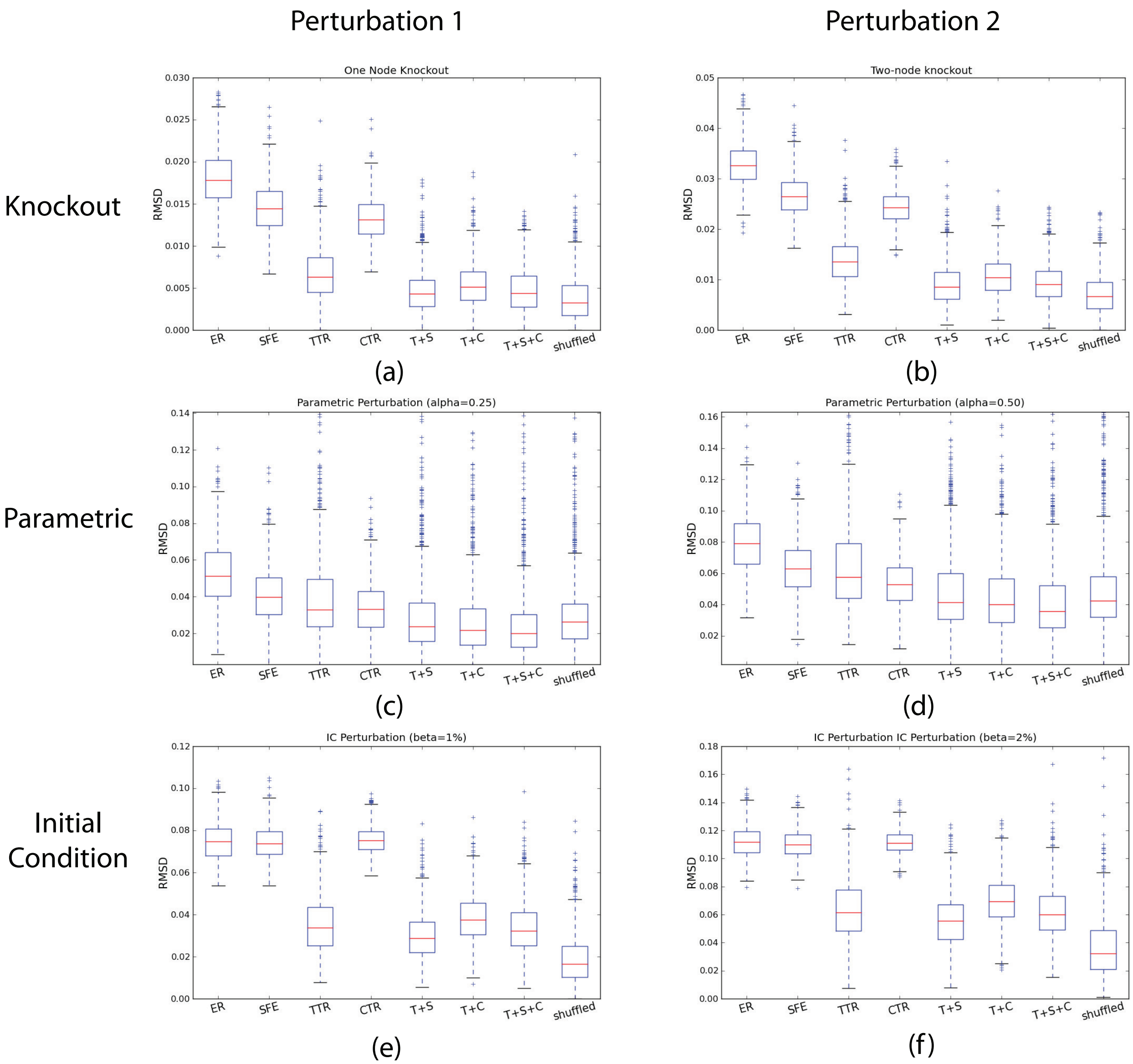

Figure S1: **RMS Deviation plots for *E.coli* ensembles**: We plot the RMS deviation for different network ensembles under knockout ((a): one-node knockout and (b): two-node knockout), parametric ((c): $\alpha = 0.05$ and (d): $\alpha = 0.10$) and initial condition ((e): $\beta = 1\%$ and (f): $\beta = 2\%$) perturbations for two perturbation magnitudes. The plots manifest that the relative ordering of the robustness profiles is invariant of the specific parameter choice and definition of robustness.[Abbreviations: T=TTR, S=SFE and C=CT]

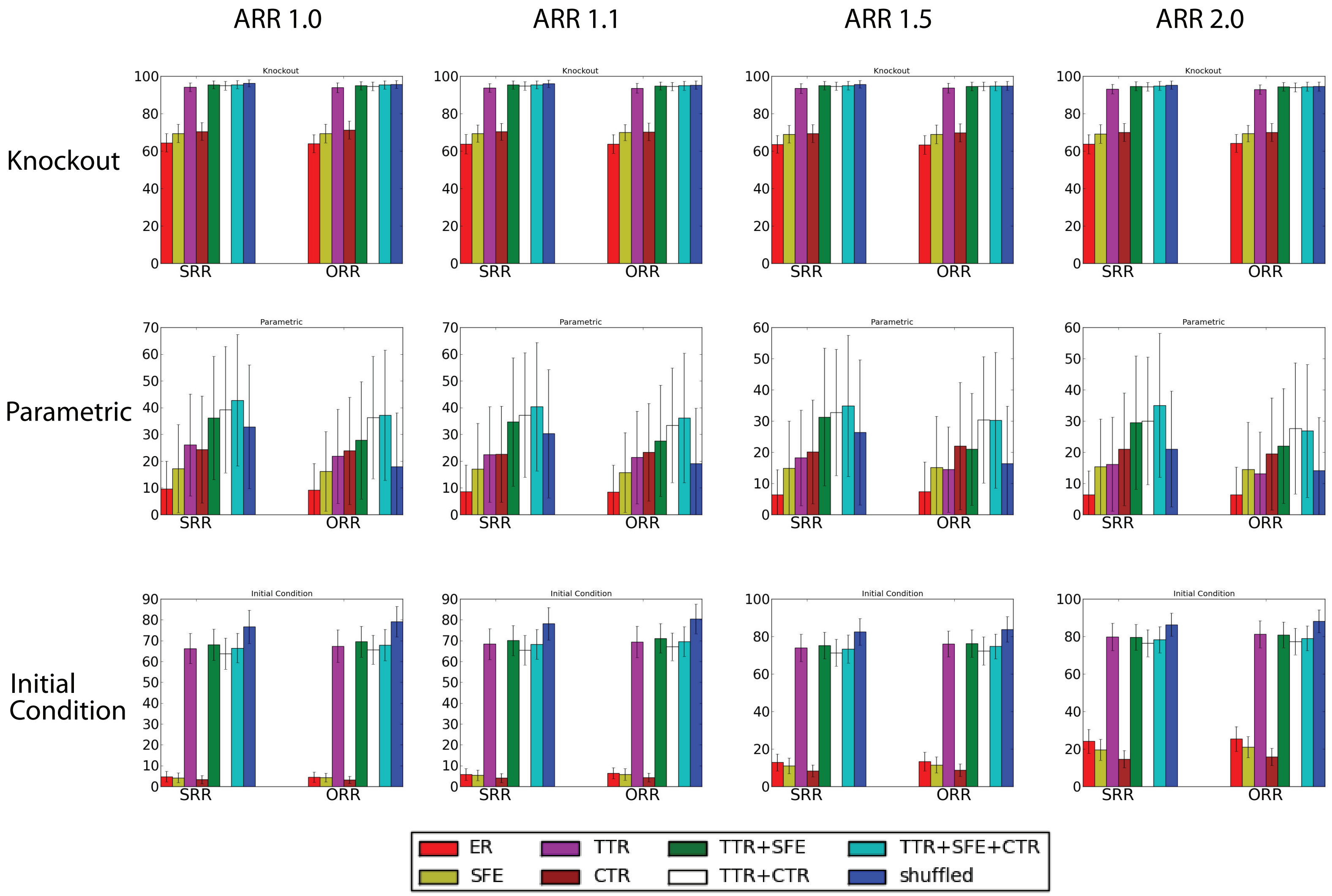

Figure S2: **Robustness profile for the *E.coli* ensembles for different Activation-Repression ratio (ARR)**: Each column represents an activation-repression ratio value used to generate the networks in all the ensembles contained, each row represents different types of perturbation. The plots manifest that the relative ordering of the robustness profiles is largely invariant of the specific ARR of the network

3.4 Partial parametric perturbation has similar robustness profile trends as the full parametric perturbation

We define parametric perturbation as perturbation of all the edges in a network by a small random amount. Here we verified that the relative contribution of the topological features remains similar if we perturb the weights of only a subset of the edges instead of perturbing all edge weights. Figure 3 reports the robustness profiles resulting from 10% edge perturbation. The relative trends of the robustness profiles among different ensembles remain almost identical which shows that the robustness ordering of the ensembles for parametric perturbation is independent of the precise definition of parametric perturbation.

3.5 Qualitative Effect of TTR on robustness is independent of other preserved properties

In Figure 2 of the main paper, we computed the robustness of networks that varied only in the number of transcription factors (TF) but constrained all other properties to assume their respective values observed in an ER network to show the positive effect of decreasing transcription factor-target ratio (TTR) on knockout and initial condition robustness. Here we verify that this positive effect persists in the TF-varied networks which differ in their number of transcription factor but conserve the cross-talk ratio or the SFE degree distribution from real biological networks. In figure S4, we plot the effect of varying the number of transcription factor in the system on its robustness for both cross-talk (Figure S4 (a)-(c)) and SFE (Figure S4 (d)-(f)) which manifests that the effect of TTR is independent of the other constraints imposed on the network.

3.6 In-degree out-degree combination for *E. coli* and Yeast networks

In figure S5, we present the in-degree vs. out-degree scatter plot for the reference yeast and *E. coli* networks. As described in the main text, this in-out-degree combination results in less robustness against parametric perturbations but more robustness against initial condition perturbation.

3.7 Basin of attraction analysis

Here we analyze how the number of transcription factors impacts the dynamical complexity of the network. We constructed an ensemble of networks consisting of 1000 networks having 100 nodes and 216 edges each (preserving the average degree of the *E.coli* transcriptional network). We then applied 1000 random initial state configuration on these networks and recorded the output states the networks converge to. The set of states where a network can reach through dynamical simulation defines the attractors of the system and the number of different initial conditions associated with a particular attractor state is an estimation of the size of its basin of attraction of the system. In Figure S6, we show that as we increase the number of TFs in a system, the mean and variance of the number of attractors increase, an observation that implies network complexity increases with the increase of number of TFs.

How does the complexity of networks impact robustness ? To answer this question, we computed the knockout and initial condition robustness (in terms of SRR) for the network ensemble and plotted the robustness values against the number of attractors and maximum basin size for an attractor in the system in Figure S7 (and Figure 4 of the original text). Both knockout and initial

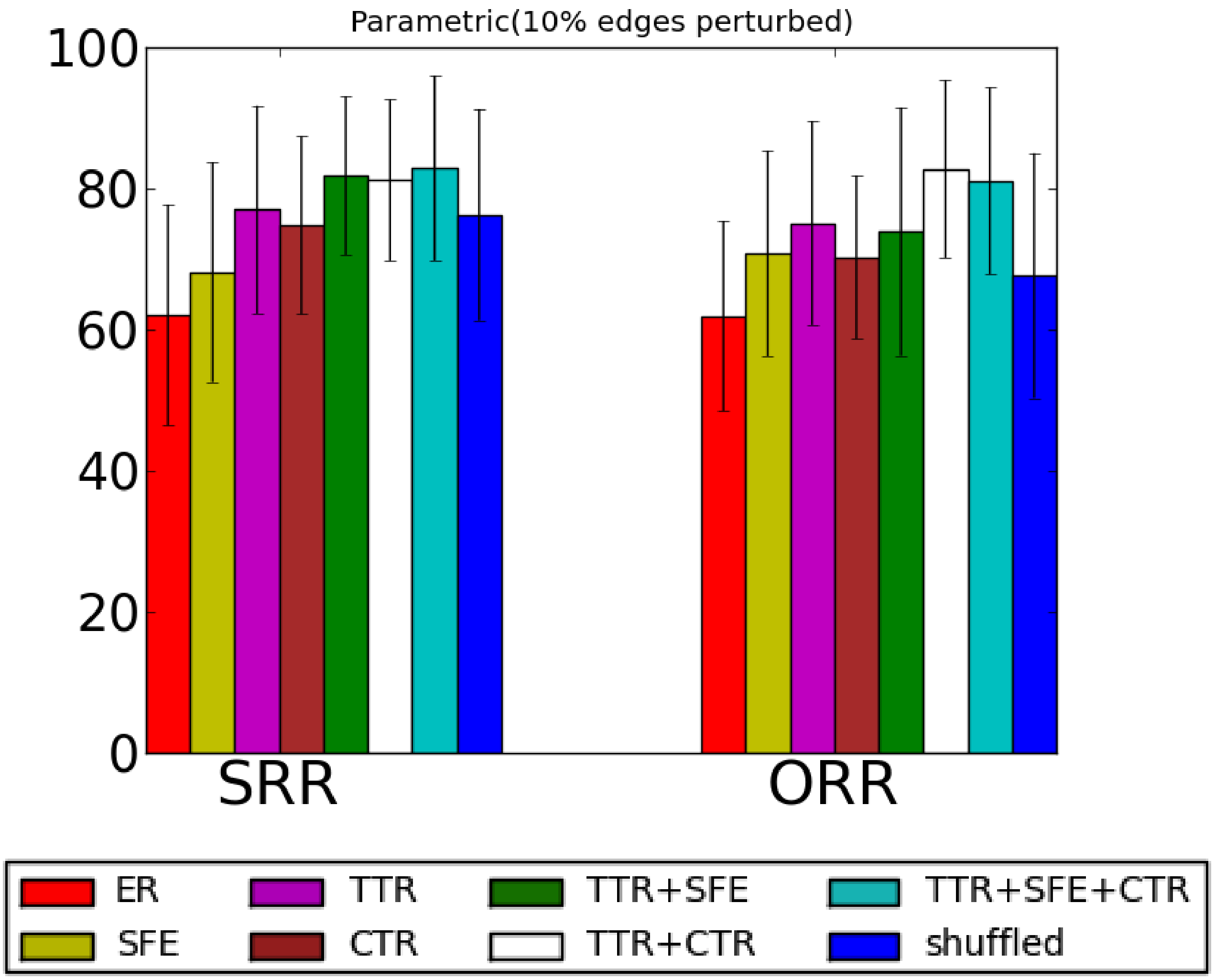

Figure 3: **Partial parametric perturbation**: Robustness (SRR and ORR) plots of the *E.coli* ensembles for partial parametric perturbations (10% of the edges perturbed). As expected, the partial parametric perturbation scales up the heights of the bars. However, the relative trends of the robustness profiles among different ensembles remains almost identical which shows that the robustness ordering of the ensembles for parametric perturbation is independent of the precise definition of parametric perturbation.

condition robustness were found to be strongly influenced by the basin size and attractor count, although initial condition robustness showed the stronger correlation coefficient values.

3.8 Correlation between features

In general, constraining one property of a network may impact the values of other properties of the network. For example, if all the genes in a transcriptional regulatory system act as transcription factors, then the cross-talk ratio will be 1.0, as every edge in the system connects two transcription factors and so, every edge, according to our definition, work as a cross-talk edge. However, it is possible to make the first-order degree-based features we considered independent of one another. We chose implementation schemes that ensured that fixing one feature did not alter the distribution of the other two features. When we impose a property set S on an ensemble E, the rest of the properties $U - S$ (where U is the universal property set) are taken from the ER network ensemble. For example, when CTR property is not preserved, the TFs are free to connect with any gene in the network, irrespective of their TF or non-TF status. This ensures that the mean CTR of the resulting ensemble is 1.0, as observed in ER networks. When the SFE property is not preserved, the endpoints of the edges are selected randomly from the appropriate set of transcription factors or targets without taking into consideration of their degree, resulting in a Poisson degree distribution

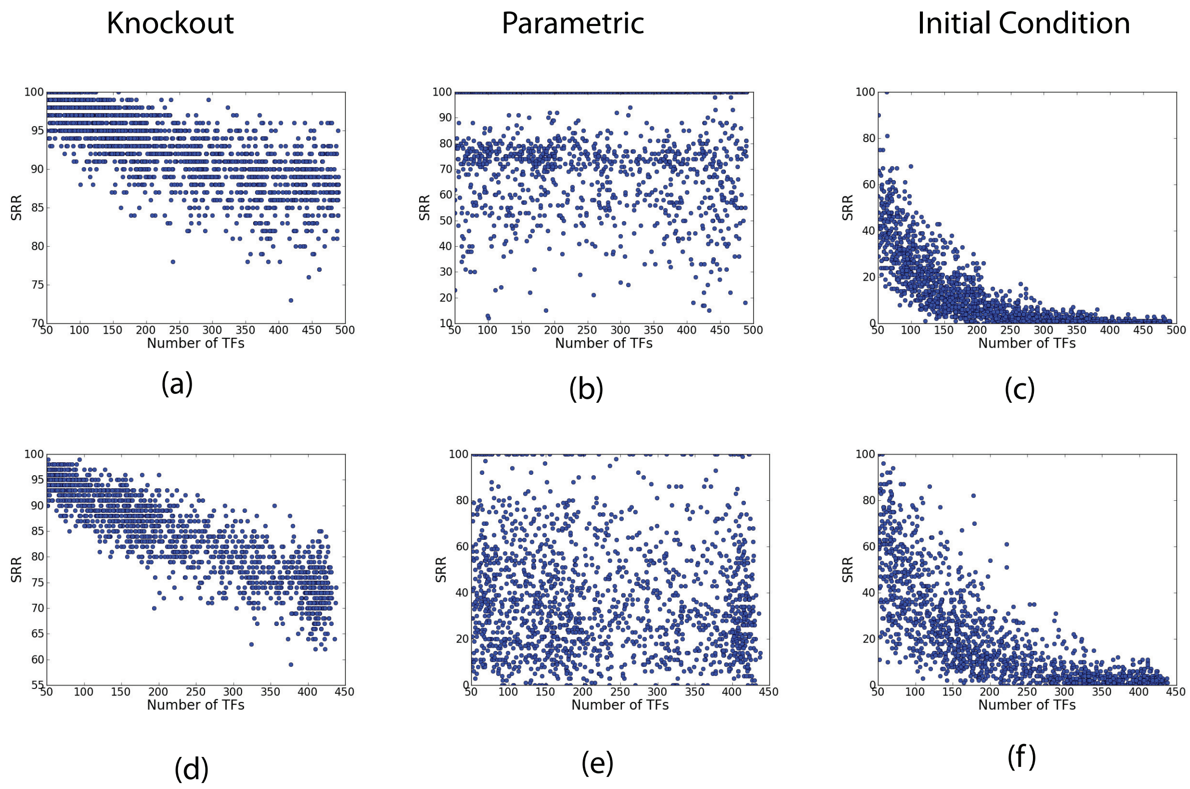

Figure S4: **Effect of TTR on robustness**: Robustness (SRR) is plotted against a wide range of transcription factor count. All the plots are for networks having same number of nodes and edges as the yeast reference network. Plots (a)-(c) represents networks where the cross-talk of the yeast reference have been preserved while plots (d)-(f) represents networks with the SFE distribution of the yeast network is preserved. Comparing with Figure 2 of the main text, we observe that the effect of TTR on robustness is independent of what other properties, if any, have been preserved.

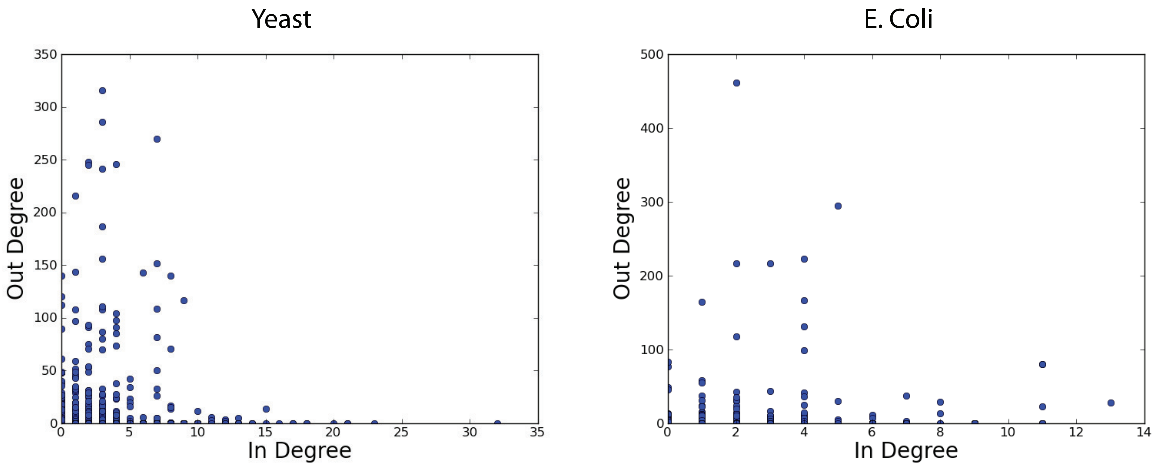

Figure S5: **In-out degree combination of the yeast and *E. coli* networks**: For each node in the regulatory network of yeast and *E. coli*, we plot the out-degree against the in-degree of the node.

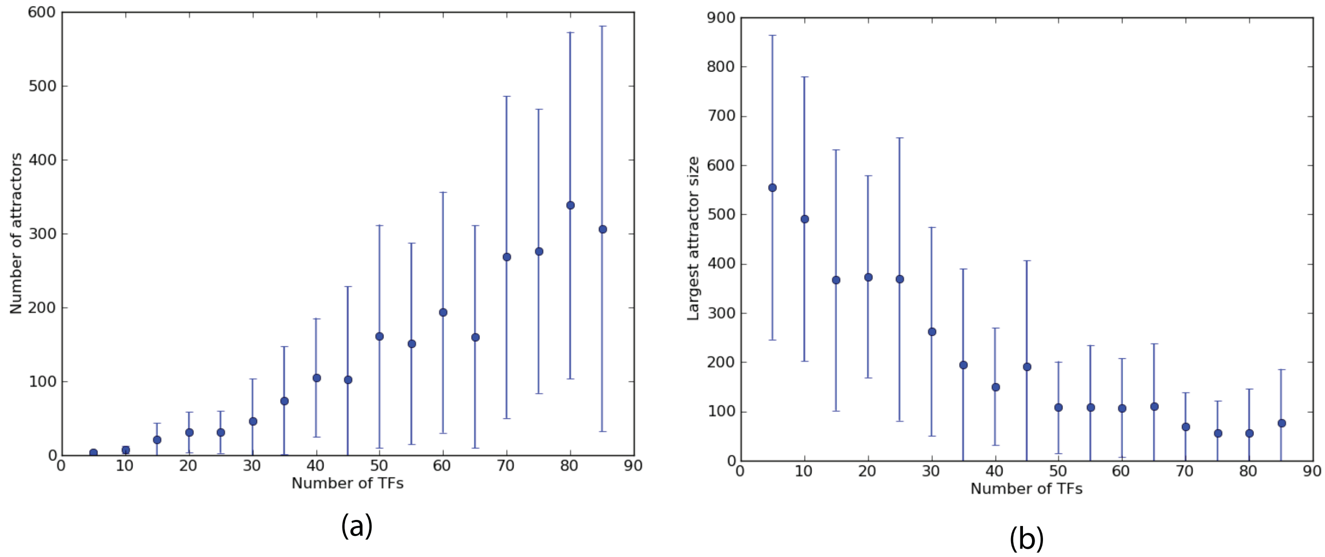

Figure S6: **Increasing the number of transcription factors increases network complexity**: Results of basin analysis with 1000 different random initial condition on 100 node and 216 edges networkw where the number of TFs have been varied from 5 to 90 and the number of attractors (plot (a)) and the largest attractor size (plot (b)) in terms of size of the basin of attraction is reported. As we increase the number of TFs, the number of attractor increases (and the variance) and the size of largest attractor (and the variance) decreases.

expected in a ER network. When the TTR property is not preserved, the expected number of

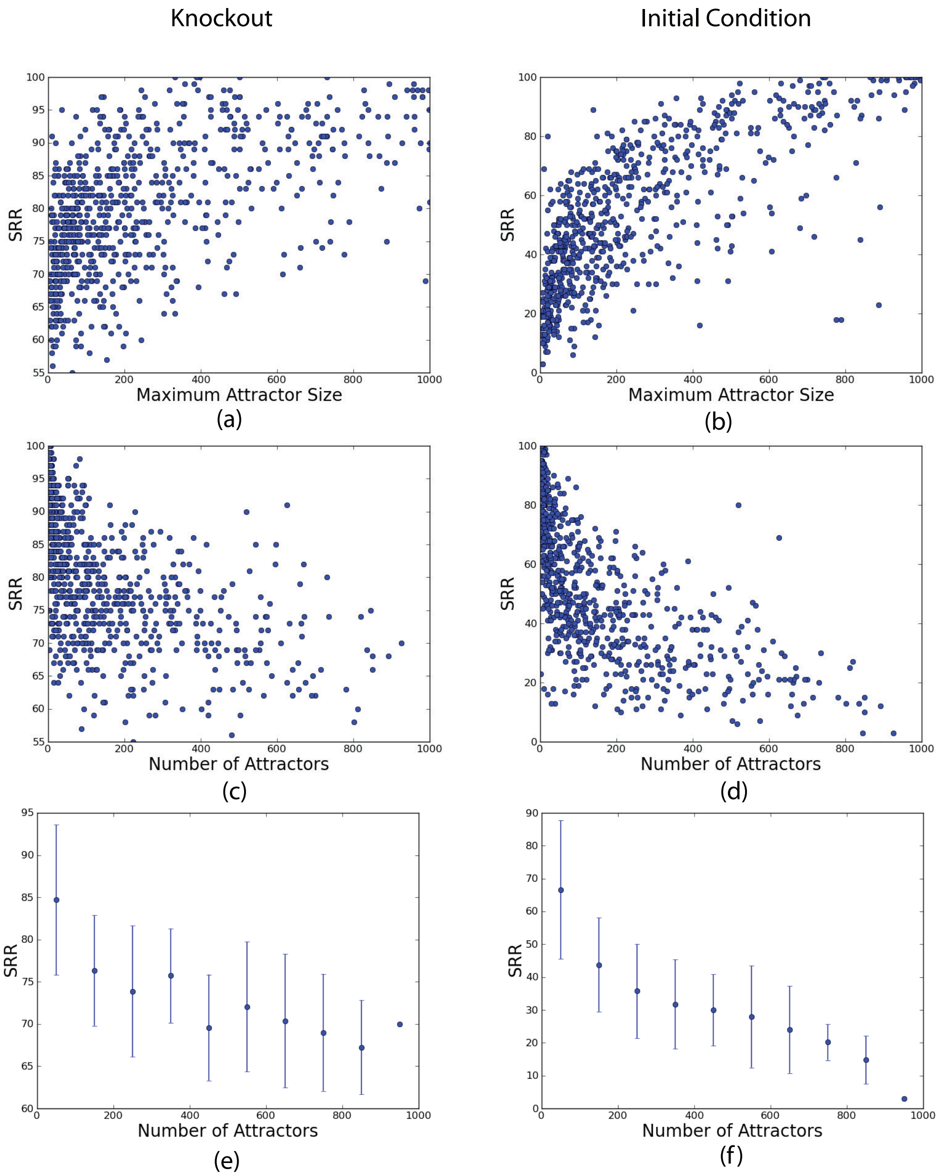

Figure S7: **Robustness decreases with the increase of number of attractors**:Knockout (plots (a),(c),(e)) and initial condition (plots (b),(d),(f)) robustness (vertical axis) is plotted against maximum attractor size (plots (a)-(b)) and number of attractors (plots (c)-(f)). Plots (e)-(f) are the binned versions of plots (c)-(d). Both knockout and initial condition robustness are strongly affected by the size of the basin of attraction as well as the number of attractors, although the trend is stronger for the initial condition robustness.

transcription factors in an equivalent ER network and the variance are first computed analytically and then these mean and variance values are used to draw the number of transcription factor of the network. Thus we ensure that the inter-feature correlation does not affect the ensembles.

References

[1] A.L. Barabási and R. Albert. Emergence of scaling in random networks. *Science*, 286(5439):509, 1999.

[2] N. Bhardwaj, P.M. Kim, and M.B. Gerstein. Rewiring of transcriptional regulatory networks: Hierarchy, rather than connectivity, better reflects the importance of regulators. *Science's STKE*, 3(146):ra79, 2010.

[3] N. Bhardwaj, K.K. Yan, and M.B. Gerstein. Analysis of diverse regulatory networks in a hierarchical context shows consistent tendencies for collaboration in the middle levels. *Proceedings of the National Academy of Sciences*, 107(15):6841, 2010.

[4] P. Erdős and A. Rényi. On the evolution of random graphs. *Publ. Math. Inst. Hung. Acad. Sci*, 5(1):17–60, 1960.

[5] S. Gama-Castro, V. Jimenez-Jacinto, M. Peralta-Gil, A. Santos-Zavaleta, M.I. Penaloza-Spinola, B. Contreras-Moreira, J. Segura-Salazar, L. Muniz-Rascado, I. Martinez-Flores, H. Salgado, et al. RegulonDB (version 6.0): gene regulation model of Escherichia coli K-12 beyond transcription, active (experimental) annotated promoters and Textpresso navigation. *Nucleic Acids Research*, 2007.

[6] N. Guelzim, S. Bottani, P. Bourgine, and F. Képès. Topological and causal structure of the yeast transcriptional regulatory network. *Nature Genetics*, 31:61, 2002.

[7] Y.K. Kwon and K.H. Cho. Quantitative analysis of robustness and fragility in biological networks based on feedback dynamics. *Bioinformatics*, 24(7):987, 2008.

[8] D. McDonald, L. Waterbury, R. Knight, and MD Betterton. Activating and inhibiting connections in biological network dynamics. *Biology Direct*, 3(1):49, 2008.

[9] H. Yu and M. Gerstein. Genomic analysis of the hierarchical structure of regulatory networks. *Proceedings of the National Academy of Sciences*, 103(40):14724–14731, 2006.
